# Development of an Oil-in-Water Self-Emulsifying Microemulsion for Cutaneous Delivery of Rose Bengal: Investigation of Anti-Melanoma Properties

**DOI:** 10.3390/pharmaceutics12100947

**Published:** 2020-10-05

**Authors:** Farzaneh Forouz, Maryam Dabbaghi, Sarika Namjoshi, Yousuf Mohammed, Michael S. Roberts, Jeffrey E. Grice

**Affiliations:** 1 Therapeutics Research Group, University of Queensland Diamantina Institute, The University of Queensland, Woolloongabba, QLD 4102, Australia; f.forouz@uq.edu.au (F.F.); m.dabbaghi@uq.edu.au (M.D.); s.namjoshi@uq.edu.au (S.N.); y.mohammed@uq.edu.au (Y.M.); m.roberts@uq.edu.au (M.S.R.); 2 School of Pharmacy and Medical Sciences, University of South Australia, Adelaide, SA 5000, Australia; 3 Therapeutics Research Centre, Basil Hetzel Institute for Translational Medical Research, The Queen Elizabeth Hospital, Woodville, SA 5011, Australia

**Keywords:** Rose Bengal, self-emulsifying micro emulsion (SEME), melanoma, targeted cutaneous drug delivery, pharmaceutical excipients, cytotoxicity, MTT

## Abstract

The topical delivery route is proposed as an alternative or adjunctive approach to melanoma treatment, since the target site for melanoma treatment—the epidermal basal layer—is potentially accessible by this route. Microemulsion systems are effective delivery vehicles for enhanced, targeted skin delivery. This work investigated the effect of Rose Bengal (RB) and RB-loaded self-emulsifying microemulsions (SEMEs) on growth inhibition of human melanoma and normal skin cell monolayers, the safety of the excipients incorporated in SEMEs on human cell lines, and the in-vitro human skin penetration of RB delivered in SEMEs and control solution. Cellular toxicity was assessed by the 3-(4,5-dimethylthiazol-2-yl)-2,5-diphenyltetrazolium bromide (MTT) assay, and the growth inhibitory mechanism of RB was investigated by flow cytometry using PI staining. Unloaded SEMEs caused reduced cellular toxicity compared to the surfactant excipient, Labrasol^®^. RB-loaded SEMEs increased cell growth inhibition compared to the RB aqueous solution. Flow cytometry revealed apoptotic cells after treatment with RB-loaded SEMEs, indicating that apoptosis may be one of the mechanisms of cell death. Preliminary results of multiphoton microscopy with fluorescence lifetime imaging (MPM-FLIM) analysis showed deeper penetration with greater skin concentrations of RB delivered from SEMEs compared to the RB aqueous solution. This study highlights the enhanced skin penetration and antimelanoma effects of RB loaded in a SEME system.

## 1. Introduction

Cutaneous melanoma is one of the deadliest metastatic neoplastic diseases with an increasing frequency globally. Australia is a high-risk region for development of melanoma skin cancer, and despite the extensive prevention campaigns, melanoma is still the second most commonly diagnosed cancer in the states of Queensland and New South Wales [1,2]. There is clearly a need for effective personalized therapy, particularly for early stage disease. Currently, surgical excision is the gold standard for melanoma treatment. However, topical application of antimelanoma agents might be a useful alternative or adjunctive approach [3,4,5], especially since the target site for melanoma treatment, the epidermal basal layer [6], is potentially accessible by this route. Transdermal drug delivery systems have introduced some therapeutic advantages for different skin conditions such as bypassing liver metabolism, enhancing drug release and site-targeting, as well as improving in patient compliance. However, the number of suitable drugs is limited. In particular, drugs with molecular weights over 500 do not penetrate the skin barrier in sufficient concentrations to provide the required efficacy at target sites in the skin. Many promising anticancer drugs are large and require enhancement strategies to penetrate the skin. One of the most promising approaches for enhanced topical delivery of agents for the treatment of skin cancer, including melanoma, is the use of nanoencapsulation. Various nanovesicular formulations have been utilized for this purpose, including liposomes, ethosomes, transferosomes, niosomes, and solid lipid nanoparticles [7,8,9,10,11]. In addition to enhancing drug bioavailability by helping to overcome the skin barrier, these nanosystems can enhance stability of the active compound, and aid in the formulation of poorly water-soluble (lipophilic) compounds. They may be further improved by incorporating penetration enhancers [12].

Rose Bengal (RB) (4, 5, 6, 7-tetrachloro-2, 4′, 5′, 7--tetraiodofluorescein disodium salt) is a stain that has been used as a diagnostic tool for investigating ophthalmic damage as well as for liver function. It has a good safety profile as a photodynamic agent with minimal potential side effects [13]. RB was shown to cause direct toxicity against melanoma cell lines by two independent mechanisms of cell death; firstly, through apoptosis, and secondly, following release of cathepsins into the cytosol [14]. Results from studies carried out to better understand the RB mechanism of action and its role in managing melanoma showed that intralesional RB injection induced a tumor-specific T-cell-mediated immune response [15] through activation of dendritic cells and release of high-mobility group box 1 to enhance a systemic immune response against melanoma [16].

The significant antitumor potency of RB suggests that alternative approaches such as topical delivery are worth investigating, especially considering the advantages that such a convenient delivery system has over other invasive treatments such as intralesional administration. However, an effective means of topical RB delivery for melanoma targeting is lacking. Promising results from studies in which RB was used to treat other skin targets such as psoriasis [17,18] suggest that topical administration targeted to the basal epidermal layer may also be a viable delivery route for melanoma. However, as noted above, few potential anticancer drugs are suitable for skin delivery. Rose Bengal has a high molecular weight (973.7 Da) and is highly lipophilic (log P 7.6, PubChem), making it a poor candidate to penetrate the stratum corneum (SC) barrier. Therefore, an advanced delivery system that provides significant skin penetration enhancement is required to provide sufficient exposure of the melanoma cells to the antitumor agent. 

Compared to other nanovesicular systems, emulsions are simple to prepare and are effective in enhancing permeation into the skin by (i) providing a high solubilization capacity for both lipophilic and hydrophilic compounds, thereby increasing the loading capacity and dose application of the formulation; (ii) facilitating a large area of surface contact with the stratum corneum through their large surface area and good skin contact combined with their occlusive nature; (iii) having the possibility of direct permeation enhancement effect on the stratum corneum lipid structure due to their oil and surfactant content [12]. Such permeation enhancing components can be specifically chosen to maximize the response. For example, our group showed significantly improved flux of two model drugs, caffeine and naproxen, across the epidermal layer of human skin in vitro when applied in a nanoemulsion containing oleic acid or eucalyptol as skin permeation enhancers [19]. Self-emulsifying emulsion systems can also be produced and the process of self-emulsification is directly influenced by the proportion of oil/surfactant pairing, concentration, ratio, and the temperature at which self-emulsification occurs and only particular combinations of pharmaceutical excipients are able to lead to effective self-emulsifying systems [20]. The self-emulsification ability of some surfactants leads to the formation of supramolecular structures with multilayers of emulsions which demonstrated potential for in vivo applications in topical treatments. These multilayer emulsions showed enhanced skin drug delivery with long-lasting release, and thus, longer therapeutic effects compared to the conventional emulsion systems [21]. In this work, we chose SEMEs (self-emulsifying microemulsions) as the delivery system for RB.

Many drug delivery systems, including emulsions, include ingredients such as Labrafac^®^ and combinations of nonionic surfactants such as Labrasol^®^ and Transcutol^®^ [22,23], which increase the bioavailability of active compounds by enhancing the solubility of lipophilic compounds and the permeation of active drugs. However, they are not considered to be completely nontoxic [24] and require assessment. Surfactants are the most commonly used solubilizing and stabilizing components in topical formulations and are known to cause varying degrees of adverse effects, including skin dryness, itchiness, and irritation [25,26,27]. A common strategy for in vivo, preclinical safety evaluation studies to ensure general safety of topical products is to collect accurate toxicity information of all the excipients before incorporating them into the final products for human use applications [28,29]. Among the most widely used in vitro endpoint biological tools to predict toxicity of an ingredient in cell culture are the viability assays based on measuring cell proliferation rate [30], commonly assessed by the MTT test.

The mechanisms of cell toxicity caused by chemical and pharmaceutical agents continue to be extensively investigated. Apoptosis, a programmed cell death, is a critical process involved in various normal cellular development and function as well as chemical-induced cell death. Understanding the mechanism of cell death induced by chemical agents at the molecular level is very important for developing potential cancer therapies. Apoptotic cell death can be studied through analysis of the cell cycle and cell cycle arrest [31]. Toxic compounds may trigger cell death by releasing cellular elements that damage DNA and unrepaired DNA damages, causing cell apoptosis [32].

This work focuses on using RB as a cytotoxic agent incorporated into self-emulsifying microemulsions to target melanoma in a cell culture model. The dose-dependent action of RB was evaluated to provide a proof of concept for the antimelanoma effects of RB in vivo. Cytotoxicity of the raw ingredients and the blank emulsions was tested to generate a toxicity profile for safe cellular application and to determine the effects of emulsification on the toxicity behavior of the raw material. The antiproliferation properties of RB were evaluated in different loading systems to compare their efficiency of use as targeted therapy for treating melanoma using RB’s intrinsic cytotoxicity. The human skin penetration of RB loaded in different systems was examined to prove the feasibility of topical application using a microemulsion delivery system. We believe that topical administration of RB loaded in an O/W self-emulsifying microemulsion could improve the concentration and depth of RB delivery into the melanoma skin site during early stage tumor development.

## 2. Materials and Methods

### 2.1. Chemical and Reagents

Rose Bengal sodium salt (RB), 3-(4,5-Dimethylthiazol-2-yl)-2,5-diphenyl tetrazolium (MTT), Propidium iodide (PI), Triton X-100, and dimethylsulphoxide (DMSO) were purchased from Sigma–Aldrich (St. Louis, MO, USA). RPMI 1640, Dulbecco’s modified Eagle’s medium (DMEM), fetal bovine serum (FBS), phosphate buffered saline (PBS), trypsin–EDTA solution (170,000 U/l trypsin and 0.2 g/L EDTA), and penicillin–streptomycin solution (10,000 U/mL/L penicillin and 10 mg/mL streptomycin) were purchased from Gibco^®^. The culture flasks and multiple-well plates were from Corning^®^ Costar^®^. All the reagents and solvents were analytical grade.

### 2.2. Surfactants and Other Excipients of the Self-Emulsifying Microemulsions (SEMEs)

Labrasol^®^ (caprylocaproyl macrogol-8-glyceride/caprylocaproyl polyoxyl-8 glycerides), Transcutol CG^®^ (diethylene glycol monoethyl ether), and Labrafac^®^ (propylene glycol dicaprylocaprate) were purchased from Gattefosse (St. Priest, France). Propylene glycol and ethanol were purchased from Sigma–Aldrich^®^ (St. Louis, MO, USA). All excipients were used as provided without further purification. Ultrapure water quality was obtained from Milli-Q^®^ purification system and was used in this study.

### 2.3. Preparation of SEMEs

Self-emulsifying combinations have been formulated by the water and oil dilution method with commonly used surfactant and cosurfactants in self-emulsifying drug delivery systems [33]. Components were mixed at 37 °C with constant agitation of magnetic stirrer. The applied concentrations of active drug were dissolved in the systems at room temperature by constant agitation. To assess any signs of phase separation, the mixtures were equilibrated for a minimum of 24 h.

For the surfactant and cosurfactant mix (S-Mix), a *v/v* ratio of 3:1 surfactant to cosurfactant was selected as the most appropriate mixture to prepare self-emulsifying systems.

The compositions and percentages of SEMEs constituents are shown in the Supplementary Materials, (Table S1). 

### 2.4. Cell Culture

Human melanoma cell lines (WM164, WM1366, and D24), spontaneously immortalized normal human keratinocytes (HaCaT), and primary skin fibroblast cells were kindly provided by A/Prof. Helmut Schaider’s laboratory (University of Queensland Diamantina Institute). The cells were grown in RPMI medium (+ L-glutamine) supplemented with 5% (*v/v*) FBS and 2% penicillin/streptomycin—except for the fibroblast cells which were cultured in DMEM. Cells were routinely grown in culture flasks and maintained at 37 °C in a humidified 5% CO_2_ atmosphere. The culture media was replaced with fresh media every two to three days. Cells were detached using trypsin–EDTA when they reached approximately 80% confluence to seed or subculture. A low passage number (6–12) of primary fibroblast was used during the experiments, and regular microbial contamination testing was done.

For the 72-h MTT assay, cells were seeded into flat bottom 96-well cell culture plates in 100 μL of complete culture medium at the initial density of 8000 cells/mL. Cells were incubated under 5% CO_2_ at 37 °C for 24 h for adhering to the wells and medium was then replaced with 100 μL of fresh medium containing treatment solution at the required final concentration. Each concentration was tested in 3 replicates in 3 independent studies and control cells were exposed to the culture medium only.

For other MTT and cell cycle studies, cells were treated when they reached subconfluent density (70–80%).

### 2.5. In Vitro Cell Viability Assays: MTT Assay

To exclude any toxic effect of the blank SEMEs and their constituents on human skin cells, an MTT cell viability test was performed based on the original protocol [34]. In this assay, the yellow tetrazolium salt MTT is reduced to insoluble purple formazan crystals by metabolically live cells.

After 60 min incubation of treated cells, the excipient-containing medium was removed, and 100 μL of MTT in PBS (5 mg/mL) diluted 1:10 in medium was then added. Plates were further incubated in the dark for 2–3 h, after which time the wells were emptied and the purple formazan was dissolved by addition of 200 μL of DMSO to each well. Plates were located in a shaker for 5 min at room temperature and the absorbance of the solutions in the wells was measured at 570 nm using a microplate reader (Multiskan™ FC Microplate Photometer). The impact of each treatment was determined as the percentage of reduced tetrazolium salt against the control cells (untreated cells with medium only).

### 2.6. Inhibitory Effect of Active Agent in Different Delivery Systems

The MTT cell viability assay was also used as a proxy measure for cell proliferation. Cells were plated in 96-well culture plates, at a density of 8000 cells per well in 100 μL of medium and incubated for 24 h. Treatment solutions of active drug in milli-Q^®^ water or incorporating in SEME were prepared in cell culture medium immediately before use and added in a volume of 100 μL at desired final concentrations. After a specific period, cell viability was evaluated by MTT assay. All experiments were performed in triplicate. 

Cell proliferation inhibition in cells treated with various concentration of RB aqueous was confirmed using light microscopy.

### 2.7. Intracellular Uptake of the Active Agent

The cellular uptake of the active agent was measured after treatment for 24 h. Melanoma cells or normal human keratinocytes were grown in 6-well culture plates until they reach 70–80% confluence. The cells were exposed to 50-µM RB in different delivery systems and untreated cells were used as control. At the end of the treatments, the culture medium with floating cells was collected in tubes. Attached cells were washed twice with PBS, collected with a scraper, and added into the tube containing the floating cells. Tubes were centrifuged at 2000× *g* for 10 min. The cell pellet from each tube was lysed in 2 mL of distilled water containing 0.2% Triton-X, followed by sonication. The absorbance at 550 nm were measured with a microplate reader. The absorbance of untreated cells was used as background and subtracted from the readings.

### 2.8. Cell Cycle Analysis by Flow Cytometry

Apoptotic cells were detected using Propidium Iodide (PI) staining of treated cells followed by flow cytometry to detect the sub-G_1_ peak. It has been reported that small segments of DNA caused by DNA fragmentation can be eluted following incubation in a hypotonic phosphate-citrate buffer. When stained with a quantitative DNA binding dye such as PI, cells that have lost DNA will take up less stain and will appear to the left of the G_1_ peak [32,33].

Briefly, melanoma cells were cultured overnight in a 6-well plate to subconfluence and treated with RB (50 µM) for 24 h. Floating and adherent cells were harvested by a trypsin-based method and fixed by ice cold 70% ethanol. Cells were incubated at 4 °C for at least 24 h in the dark and stained using 70 μM Propidium Iodide in 500 µl of a 38-mM hypotonic tri-sodium citrate buffer supplemented with 5 μg/mL RNase A before flow cytometric analysis using a FACScan.

### 2.9. Evaluation of Cell Viability

The cytotoxicity at each excipient concentration was expressed as a percentage of viability against the untreated control wells (the mean optical density of untreated cells was set at 100% viability) to construct a dose-dependent cytotoxicity plot.

The percentage viability was calculated as follows (Equation (1)):

(1) $$\%\;\mathrm{Viability}=\;\frac{\mathrm{Mean}\;\mathrm{absorbance}\;\mathrm{of}\;\mathrm{treated}\;\mathrm{wells}}{\mathrm{Mean}\;\mathrm{absorbance}\;\mathrm{of}\;\mathrm{control}\;\mathrm{well}\left(\mathrm{no}\;\mathrm{excipients}\right)}\;\times 100.$$

The cytotoxicity of the excipients and formulations (treatments) was expressed as an IC_50_ value (concentration causing 50% death of the cell population) [34]. The IC_50_ value for each treatment concentration was calculated from the dose–response curves plotted in GraphPad Prism 7 (GraphPad Software Inc. California, USA) with log (treatment concentration) on the x-axis and percentage survival on the y-axis. The IC_50_ value was obtained by fitting the dose–response curve with a nonlinear regression curve (Dose-response-inhibition) on GraphPad Prism 7. Results were displayed as mean ± standard deviation (SD). All IC_50_ values were expressed as *v/v* (%).

## 3. Results

The visual properties of the microemulsions recorded against the addition of the applied surfactant mix in Ternary phase diagrams (Figure S1), Droplet Size and Zeta Potential Determination (Table S2), and Rheological measurements (Figures S3–S5) are provided in Supplementary Materials.

### 3.1. In Vitro Cell Viability Assay of SEMEs and Its Components

**Figure 1:**
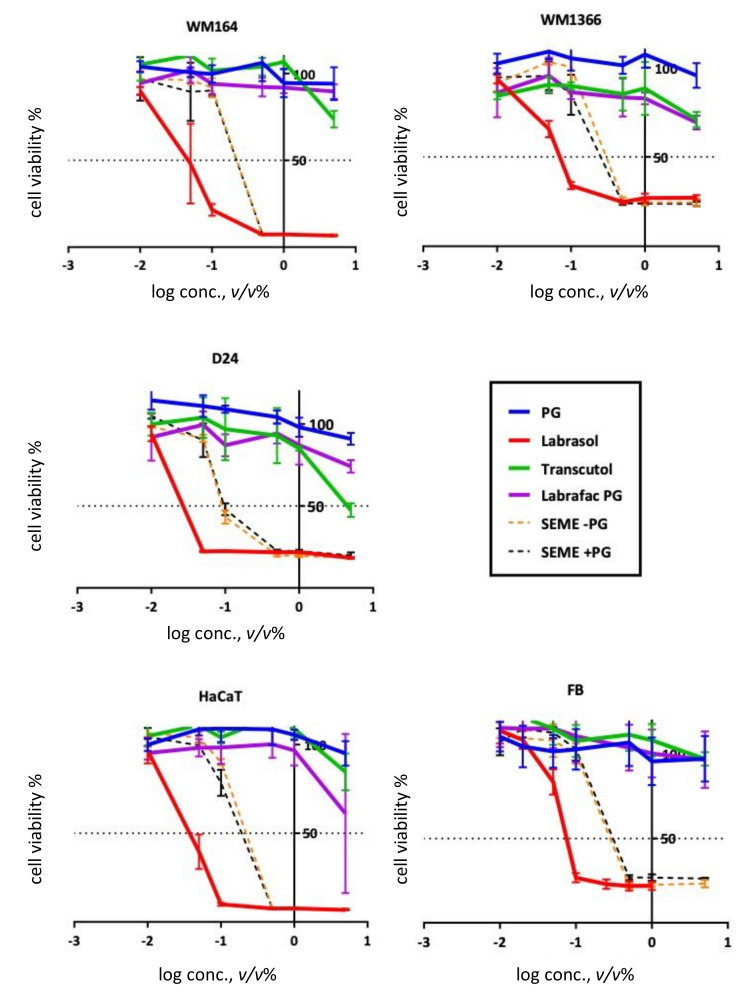
Toxicity assay. MTT assay after 1 h by MTT for different concentration of the excipients and self-emulsifying microemulsions (SEMEs). Error bars show Mean ± SD for *n* = 5.

Cytotoxic properties of the excipients were evaluated by MTT assay. This assay showed various sensitivities after a 60-min exposure of the cancerous and noncancerous cell lines to different tested concentrations. Dose–response toxicity curves for SEMEs and the excipients in all cell lines are illustrated in Figure 1 and the related IC_50_s are shown in Table 1.

Wild type melanoma cells (Table 1) showed the greatest sensitivity to the treatments among the other two cancerous cell lines with IC_50_ values ranged from 0.03–92.16 *v/v* (%). Two noncancerous cell lines, HaCaT and fibroblasts, showed reduced sensitivity to treatment with IC_50_ values ranging from 0.04–6.35 *v/v* (%) and 0.08–52.03 *v/v* (%), respectively. Labrasol^®^ showed the greatest cytotoxicity effect on all cell types with IC_50_ values ranging from 0.03–0.11 *v/v* (%). However, Labrasol^®^ incorporated into the formulation systems (the same applied concentration *v/v* (%) as in single application) showed lower cytotoxicity than a single treatment with Labrasol^®^. The formulation reduced the toxic effects of this excipient on the cell lines by up to 3-fold.

### 3.2. Treatment with Rose Bengal Aqueous Solution Induced Melanoma Cancer Cell Death In Vitro

Considering the results of SEMEs cytotoxicity test (Table 1)**,** nontoxic concentrations of the SEMEs (<IC_50_) were selected for RB loading, and for all the cell treatments in this study. 

**Figure 2:**
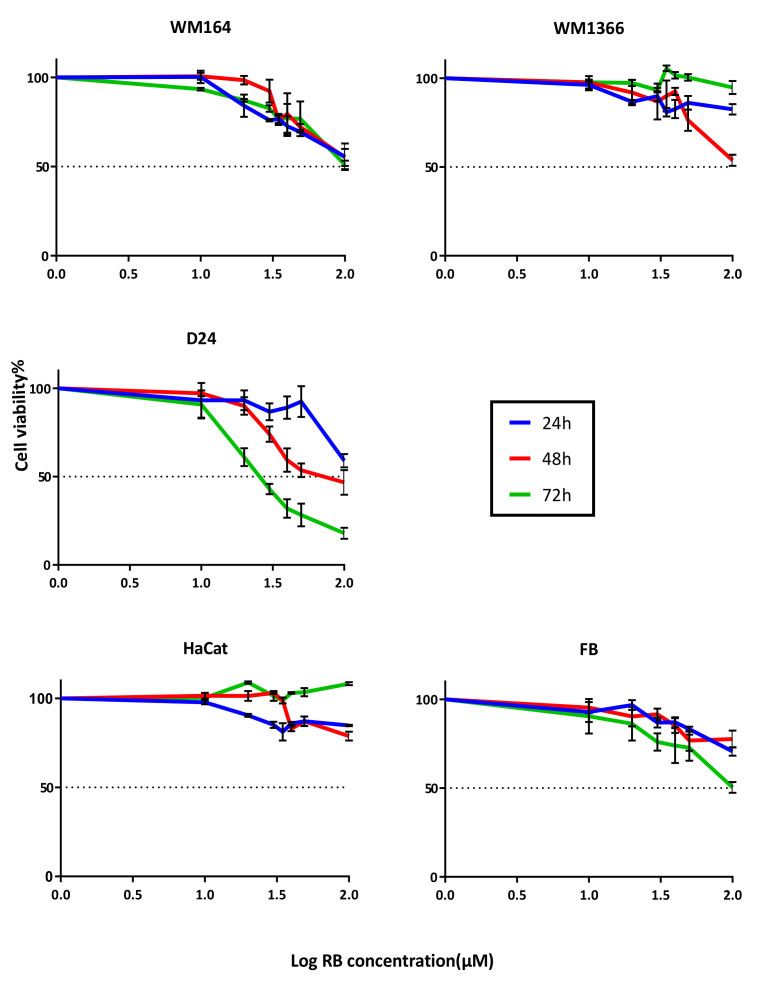
Cytotoxicity of RB aqueous solution in various cell lines. Treatment for up to 72 h. Error bars show Mean ± SD of 2 independent experiments. Each experiment was done with a minimum of three replicates.

The dose-dependent response curves for RB aqueous solutions are shown in Figure 2. Cancerous cell lines were more sensitive to the treatment than the two noncancerous cell types. The D24 cell line was the most sensitive, with lower IC_50_ values compared to the other cancerous cell lines. Treatment caused less toxicity on HaCaT than fibroblasts and both normal skin cell types showed higher IC_50_ values than the melanoma cell types after exposure to various concentrations of RB treatment (Table 2).

**Figure 3:**
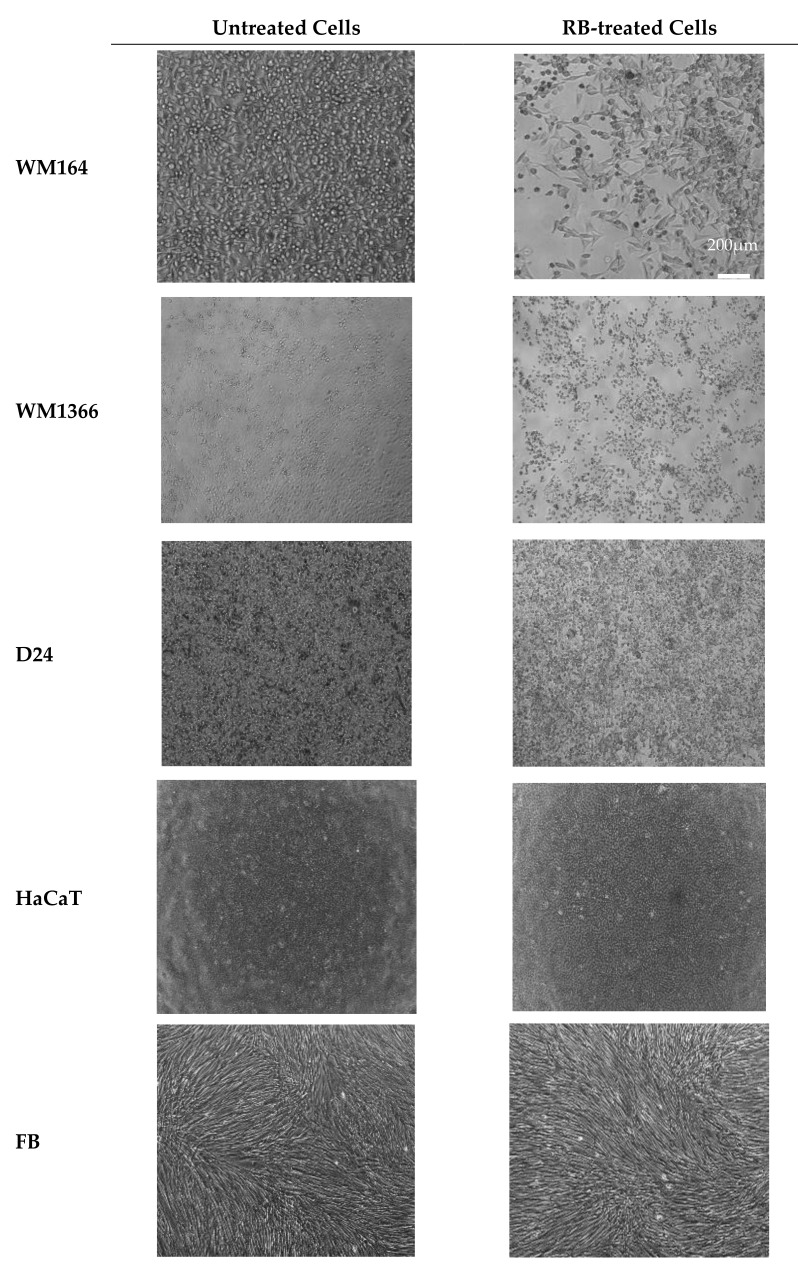
Light microscopy images of the cells. Untreated cell lines in culture and RB-treated cells after 48 h treatment with an IC_50_ concentration of RB. Bright field images are viewed under low power (4× objective). Treated melanoma cells (WM164, WM1366, and D24) appeared rounded, shrunken, and damaged. Many of the dead cells were detached from the plate and floating in the media. HaCaT cells only showed some morphological changes but remained attached to the surface and Fibroblast cells remained intact. Scale bar = 200 µm.

The cytotoxicity effect of RB aqueous solution was supported by the bright field microscopy images of live cells after exposure to the treatment with IC_50_ concentrations in all the tested cells and compared to the untreated controls (Figure 3). Treated melanoma cells (WM164, WM1366, and D24) appeared rounded, shrunken, and damaged. Many of the dead cells were detached from the plate and floating in the media. HaCaT cells only showed some morphological changes but remained attached to the surface, and fibroblast cells remained intact.

### 3.3. Inhibitory Effect of Rose Bengal in Different Delivery Systems

The antimelanoma activity of RB was tested when it was incorporated in aqueous solution and in microemulsion formulations (SEME+PG and SEME–PG). The cells were treated with various concentrations of RB, while the vehicle concentration was kept constant. 

**Figure 4:**
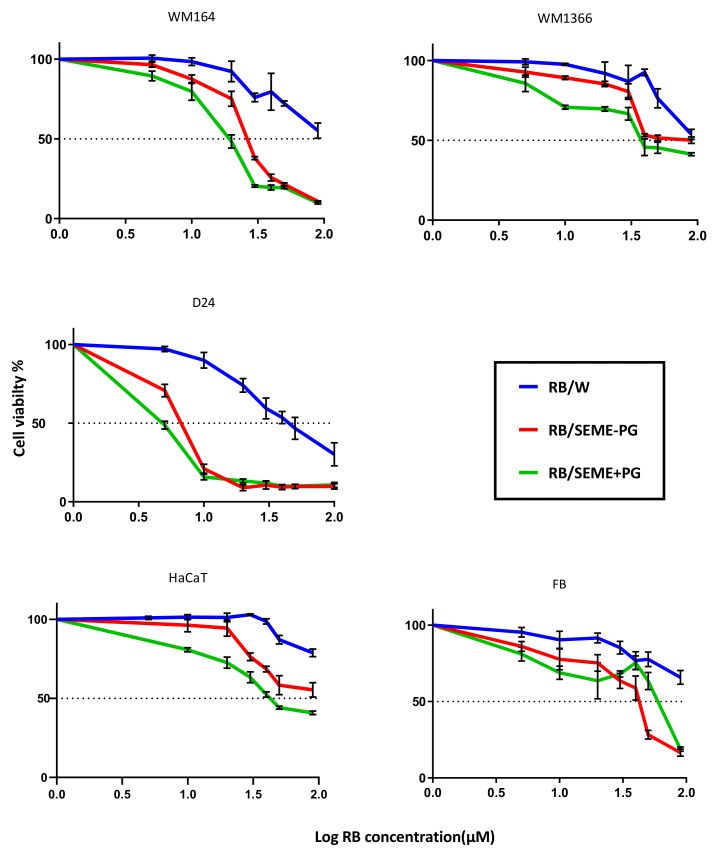
Dose-dependent response of RB and encapsulated RB. Cellular response to the treatment with RB and encapsulated RB after 48 h of incubation observed in (**A**) WM164; (**B**) WM1366; and (**C**) HaCaT cell lines. Average cell viability ± SD plotted (*n* = minimum of 4).

The cytotoxic properties of RB in aqueous solution and in SEMEs were evaluated by MTT assay after 48-h exposure, and the IC_50_ values of RB solution was compared with RB incorporated in the formulations (Figure 4 and Table 3). 

All treatments generated a dose–response curve. The results showed that the SEME formulations enhanced antimelanoma potency of RB in all cancerous cell lines, with SEME+PG being more potent than SEME–PG in reducing cell viability. Microemulsion systems increased the antimelanoma effect of RB in the normal cells, but at much higher concentrations than in the cancerous cell lines. 

### 3.4. Effect of SEME on Cellular Uptake Of The Active Agent

**Figure 5:**
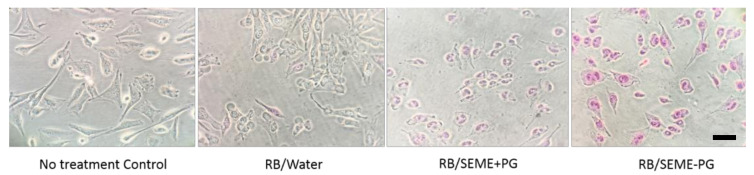
Microscopy images of RB uptake by the live melanoma cells. The cells are treated for 30 min with final concentrations of 50-μM RB incorporated in 0.02% of water, SEME + PG, and SEME–PG. The cells showed more RB uptake after treatment with SEME + PG. Images captured at 40× magnification and scale bar = 25 μm.

The melanoma cells were treated with final concentrations of 50-μM RB incorporated in 0.02% water, SEME+PG, and SEME–PG for 30 min. It is evident that cellular uptake of the active agent was enhanced in the formulations compared to the RB aqueous solution (Figure 5). Delivery of RB through the cell membrane was facilitated in the formulations, leading to a potential increased availability and anticancer potency at the cellular level. Greater melanoma cell uptake of RB was seen with the SEME formulation without PG (SEME–PG) than in the formulation containing PG (SEME+PG) for the same incubation time (Figure 5).

**Figure 6:**
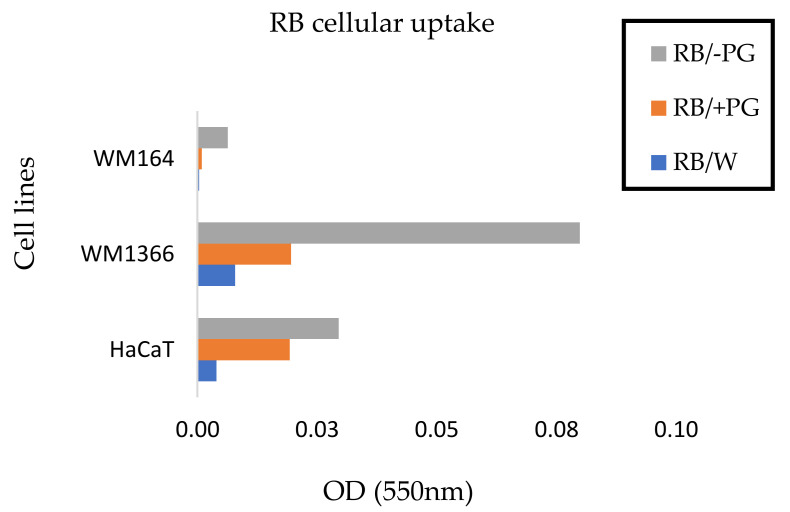
Optical density of lysates. Optical density of lysates from Melanoma (WM164 and WM1366) and normal keratinocyte (HaCaT) cells treated for 24 h with final concentrations of 50 µM RB incorporated in water, SEME–PG, and SEME + PG. RB uptake is normalized against untreated control cells. Incubation with RB incorporated in SEME–PG showed higher cellular uptake of the active agent.

RB absorbance intensity after lysing the treated cells for 24 h was plotted in Figure 6. The results are similar to those seen in the microscopy evaluation (Figure 5), with the greatest amount of RB being released after lysis of the cells treated with RB incorporated in SEME–PG.

### 3.5. Cell Cycle Analysis by Flow Cytometry

After establishing that RB decreased cell viability of human skin melanoma cells in a dose-dependent manner, the mechanism of cell death was investigated. As RB-induced cell death was evident upon exposure to concentrations >50 μM, the cells were treated with 50 μM RB in water, SEME+PG and SEME–PG for 24 h for cell cycle analysis. According to the cytotoxicity results of the SEME compositions, nontoxic concentrations (below IC_50_ values) were used in the applied treatment dosage. In cells treated with RB incorporated in SEMEs, melanoma cancer cells displayed significant reductions in the cell fraction in the S phase and increased cell fractions in the G_2_M and sub G_0_G_1_ phases, consistent with G_2_M growth arrest (Table 4 and Figure S7).

Flow analysis of the sub G_0_G_1_ phase showed increases in apoptotic cell populations when cells were treated with RB-loaded SEMEs compared to the RB aqueous solution. Melanoma cells showed increases in apoptotic populations at various levels. However, equal dosage treatments did not impact normal keratinocytes and fibroblasts. This selective cell death mechanism could be a key for an effective antimelanoma drug.

## 4. Discussion

Our results established that RB caused direct toxic effects that suppressed the growth of tested melanoma cell lines in a dose-dependent manner. In support of the anticancer properties of RB, its direct toxicity for potential anticancer therapy has been investigated through several different studies. Mousavi et al. reported that RB induces two independent mechanisms leading to cell death in melanoma cell lines—apoptotic cell death, as well as cell death through release of cathepsins into the cytosol [14]. The ability of RB to induce direct cell death has also been studied in other types of cancers such as ovarian carcinoma cells [35]. The in vitro results showed significant increasing levels of apoptosis in RB-treated tumor cells compared to untreated control cells with no effects on normal human fibroblasts. RB treatment in this study was not only mediated with generation of reactive oxygen species (ROS) but also induced apoptotic cell death [35]. Dose–responsive cell death with RB treatment, associated primarily with the autophagy cell death pathway, has also been reported in colon cancer cells [36]. Our results also showed that RB was not as effective at inhibiting cell growth of normal human fibroblasts at the same concentration that prevented the growth of melanoma cells. This is in agreement with previous studies that showed selective targeting of RB for cancer cells (melanoma, ovarian, and colon cancers), but not normal cells [35,36,37].

In designing and developing effective topical formulations, component selection is a key area. Apart from permeation properties, the toxicity of each individual component and the final product is one of the major challenges. Raw materials cannot be assumed to be nontoxic, and their potential adverse effects on skin should be evaluated before any in vivo applications [25,26,27]. In this work, for cytotoxicity evaluation of the raw materials and the blank emulsions, we used a range of cell lines of different origins—both cancerous and noncancerous—for a reliable toxicity screening. Previous studies reported significant differences in the cytotoxic effects of chemical compounds depending on the tested cell type [38,39,40,41,42].

Our observations showed that the cancerous cell lines WM164 and D24 were more sensitive to the toxic effects of the test materials than the other cancerous cell line, WM1366. Distinct sensitivity of cell lines against a chemical compound could be partially explained by the features of each cell type, which lead to a different defence mechanism against toxic exposure. Contact inhibition is a fundamental property of cells that has a direct effect on cell proliferation in culture-based experiments, and loss of this feature is associated with malignancy and tumorigenesis. Contact inhibition can cause a decrease in proliferation rates at a high cell density [43]. Our observations of the WM164 cells in culture showed that they grew in spatial layers and formed clusters, indicating their lack of contact inhibition. Uncontrolled proliferation, leading to high differentiation rates, may be the reason for the greater sensitivity of WM164 towards the toxic effects of the excipients. In contrast to the WM164, our results showed that HaCaT and primary fibroblast cells showed more resistance to the toxic materials. Several studies on cytotoxicity and phototoxicity of the compounds have indicated that HaCaT keratinocytes are a more resistant cell line than other human skin derived cell lines due to their lower growth rate resulting from contact inhibition [43]. The lower cytotoxicity responses of HaCaT cells could be attributable to the contact inhibition property, which controls the rate of proliferation and metabolic processes of the cells. In our experiments, WM1366 showed very similar morphological and phenotypic characteristics to the HaCaT cell line. These two cell lines had similar growth rates and contact inhibition properties, which could partly explain their similar toxicity responses.

Our results demonstrated a great toxicity for Labrasol^®^ in all the cell lines with the IC_50_ ranging from 0.03–0.08 (*v/v* %); while showing a lower toxicity for Transcutol^®^, with IC_50_ values ranging from 4.55 to greater than the maximum concentration used in this study, 10 (*v/v* %). Earlier studies used MTT endpoints to investigate the cytotoxicity of Labrasol^®^ and Transcutol^®^ in various cell lines, including human cervical cancer (HeLa) and human epithelial colorectal adenocarcinoma (Caco-2) cells [44,45,46]. These also reported high toxicity for Labrasol^®^, with IC_50_ values of approximately 0.2 (% *v/v*, or 2 µg/mL) and less toxic effects for Transcutol^®^ with 3.4 (% *v/v*, or 34 µg/mL) in the HeLa cell line [46].

As mentioned previously, the nonionic surfactant Labrasol^®^ is more cytotoxic than the other tested surfactants and should be cautiously employed as an additive or constituent in any topical formulations. Our IC_50_ results showed that Labrasol^®^ has the greatest toxic effects on the cell lines when applied as a single excipient. However, when the same concentration of Labrasol^®^ used alone was incorporated into the SEMEs, its toxicity was reduced by up to 3-fold. The results indicated that combining Labrasol^®^ in an emulsion system reduced the in-vitro toxicity of Labrasol^®^, thus minimizing the potential skin irritation of this material. It has been previously reported that emulsion systems could moderate skin irritancy of certain excipients. For example, an aqueous solution containing 20% propylene glycol was shown to cause irritation, whereas the same concentration of propylene glycol used as a cosurfactant in microemulsion formulations did not [19,47].

Work has been done to enhance delivery of RB transdermally using various techniques. A preclinical pharmacokinetic and safety study investigated the safety profile and topical delivery of RB in various formulations [13]. In this study, 1% RB was incorporated in hydrophilic formulations (saline and saline hydrogels), lipophilic formulations (PG, oil–water emulsion, and lipophilic liquid), and DMSO, for testing in murine and rabbit skin models. Among all the formulations, only DMSO showed significant delivery of RB to all tissue layers. Hydrophilic formulations resulted in delivery only to the epidermis, with undetectable penetration into dermis and underlying layers. At the same time, however, the epidermal delivery of RB from those hydrophilic formulations was more rapid and uniform than that from lipophilic formulations. Our results from a multiphoton microscopy with fluorescence lifetime imaging (MPM-FLIM) penetration study (Figure S6) showed that our self-emulsifying microemulsion enhanced delivery of loaded RB in greater amounts and into deeper layers of the excised human skin compared to the aqueous solution.

In recent years, microemulsion systems have gained popularity for formulating poorly water-soluble actives to enhance drug solubility, and thus availability [20]. Microemulsion systems are not only suitable for lipophilic compounds, but they are also able to enhance delivery of hydrophilic compounds, as we have seen here. Nanoemulsions have been used as carrier vehicles for hydrophilic ropinirole hydrochloride (RHCl) across rat skin. The permeation rate of drug-loaded nanoemulsion was significantly increased, while the drug in aqueous solution with or without penetration enhancers was not transported across rat skin [48].

Emulsions could also provide a compatible environment for hydrophilic compounds to facilitate their transport through multilayer skin via different delivery pathways. Water-in-oil nanoemulsions loaded with hydrophilic inulin showed a significantly higher transport rate through rat skin comparing with micellar dispersions or aqueous controls. Transport was also found to be highly dependent on the hydrophilic–lipophilic balance (HLB) of the surfactant mixture (S-Mix), so that the nanoemulsions formed with lower HLB surfactants demonstrated significantly higher rates and extents of delivery. It was suggested that the compatible nature of the vehicle with the lipophilic sebum environment of the hair follicle facilitated the transdermal delivery of the nanoemulsion loaded inulin [49].

A previous study by Qin et al. [36] investigating the cell death mechanism of RB demonstrated that 10% of RB in saline solution (PV-10) induced cell death in colon cancer cells through G_2_M phase arrest, which was not primarily by apoptosis, but predominantly through a necrotic cell death mechanism. Our data from cell cycle analysis also indicated that the treatment of melanoma cells with IC_50_ concentrations of RB aqueous solution did not induce high levels of apoptosis. However, incorporating RB into the microemulsions enhanced apoptotic populations. Further investigation is recommended to identify the other possible cell death mechanisms that might be involved.

The images for our cells treated with the same concentration of RB loaded in various formulations showed the higher drug uptake from the formulation compared to the aqueous solutions. It also showed higher RB uptake from SEME–PG formulation in incubated cells compared to SEME+PG. Previous studies have demonstrated that Labrasol^®^ [50] and other excipients [51] can play a role in efflux-mediated transport by inhibiting the drug efflux from the cell. Labrasol^®^ and Transcutol^®^, as common excipients used in Self-emulsifying Drug Delivery Systems (SEDD), have been studied individually and in combination for their influence on efflux inhibition. It has also been shown that combined use of the excipients might increase or decrease inhibition of efflux-mediated transport, and this should be considered for the optimized combination to be used in the formulation [46]. Considering these findings; our emulsion without PG appeared to be the optimal combination that inhibited efflux due to Labrasol^®^ and/or Transcutol^®^ present in the formulation. On the other hand, lower uptake of RB by the cells from SEME+PG could be due to increased solubility of the cell membrane by PG as penetration enhancer, causing RB clearance from the cells and neutralising the efflux inhibitory effect of other excipients. Studies have shown that addition of cryoprotective agents (CPAs) such as propylene glycol (PG) can decrease freezing damage but can also interrupt membrane integrity initiating leakage of intracellular constituents [52]. It has also been reported that adding CPAs causes lateral expansion of the membrane, thus, reducing the thickness of the bilayer lipids [53].

## 5. Conclusions

Of the tested formulation excipients, Labrasol^®^ was the most cytotoxic, while Labrafac^®^ was the least. All melanoma cells were more sensitive to treatments than the normal cells (keratinocytes and fibroblast cells). The WM164 melanoma cell line was the most sensitive, which could be explained by the lack of cell contact inhibition in this cancerous cell type. Incorporation in self-emulsifying microemulsions reduced the toxic effect of a single excipient in each tested cell line.

Rose Bengal in aqueous solution showed dose-dependent antimelanoma properties in the tested cancerous cell lines. Incorporation of RB into the emulsion systems increased the antimelanoma effect of this compound in melanoma cells, with less effect on normal skin cells. The microemulsions enhanced the cellular uptake of RB compared to the aqueous solution, which could be explained by the possible efflux inhibition effect of Labrasol^®^ present in the formulations. In addition, increased delivery of RB to deeper skin layers occurred when RB was incorporated in SEMEs containing PG, compared to an aqueous RB solution. The mechanism of cytotoxicity, investigated by flow cytometry, was shown to include induction of apoptosis, although other mechanisms could not be excluded.

We conclude that self-emulsifying microemulsion delivery systems are able to enhance the antimelanoma potency and targeted skin delivery of RB and show promise to be developed for noninvasive treatment of early stage cutaneous melanoma.

## Figures and Tables

**Table 1:** IC_50_ values of excipients in the tested cells. IC_50_ values on WM164, WM1366, D24, and HaCaT cell lines after 60 min of incubation. All values are expressed in mean concentration of excipients (*v/v* %) with 95% confidence interval indicated in brackets (*n* = 5). IC_50_ values above 100% *v/v* (%) were considered as not converged. (>Max Conc.; not reaching the sensitive range for the applied concentrations).

Excipients	IC_50_ (*v/v* %)				
	WM164	WM1366	D24	HaCaT	Fibroblast
PG	>Max Conc.	>Max Conc.	14.74 (very wide)	>Max Conc.	52.13 (very wide)
Labrasol^®^	0.04 (0.034–0.058)	0.11 (0.06–0.21)	0.03 (0.02–0.06	0.04 (0.03–0.04)	0.08 (0.06–0.13)
Transcutol^®^	~5.6 (very wide)	>Max Conc.	4.55 (3.1-8.2)	5.75 (very wide)	>Max Conc.
Labrafac^®^	>Max Conc.	>Max Conc.	92.16 (very wide)	6.35 (4.03-8.02)	>Max Conc.
SEME–PG	0.22 (0.18–0.27)	0.37 (0.25–0.52)	0.15 (0.09–0.23)	0.22 (0.17–0.27)	0.34 (0.25–0.45)
SEME + PG	0.21 (0.13–0.29)	0.30 (0.21–0.45)	0.18 (0.11–0.28)	0.18 (0.14–0.22)	0.39 (0.28–0.53)

**Table 2:** IC_50_ values of Rose Bengal (RB) aqueous solution.

RB Aqueous Solution	IC_50_ (µM)				
	WM164	WM1366	D24	HaCaT	Fibroblast
24 h	112.2 (92.5–147.2)	>Max Conc.	130.9 (106.6–197)	2254 (633.2–36213)	238 (181.7–356.1)
48 h	106.6 (88.9–138.8)	109.8 (93.74–139.2)	69.97 (60.2–84.67)	221.3 (155.7–465.4)	458.7 (252.1–1405)
72 h	109.1 (93–136)	~113.7 (Very wide)	27.33 (25.2–29.6)	>Max Conc.	108.2 (87.1–150.5)

**Table 3:** IC_50_ values of RB and encapsulated RB on WM164, WM1366, D24, and HaCaT cell lines after 48 h of incubation. All values are expressed in mean concentration of RB (μM) with 95% confidence interval indicated in brackets (*n* = minimum of 4).

RB/Vehicles	IC_50_ (μM)				
	WM164	WM1366	D24	HaCaT	Fibroblast
RB/W	102.8 (86.4–131.9)	98.93 (86.6–120.8)	45.6 (42.0–49.8)	152.8 (127.3–204.1)	188.4 (142.2–284.1)
RB/SEME–PG	27.03 (25.4–28.7)	69.55 (57.7–90.2)	6.73 (6.07–7.4)	86.95 (71.9–114.9)	37.2 (32.7–42.2)
RB/SEME+PG	18.58 (17.1–20.1)	47.06 (39.7–57.5)	4.86 (4.2–5.6)	49.27 (44.7–54.9)	55.95 (41.1–89.4)

**Table 4:** Percentages of apoptotic cell populations of cancerous and noncancerous cell lines after 24-h treatment with RB loading formulations. Results were derived from cell cycle histograms shown in Supplementary Materials Figure S7.

Apoptotic Cells % (Sub G_0_G_1_)	Untreated	RB/W	RB/SEME–PG	RB/SEME + PG
D24	2.54	2.66	4.79	5.83
WM164	3.94	6.69	9.12	9.50
WM1366	0.89	2.29	4.04	5.24
HaCaT	1.07	1.15	1.36	1.03
Fibroblast	0.00	0.99	0.93	0.69

## Supplementary Materials

The following are available online at https://www.mdpi.com/1999-4923/12/10/947/s1, Figure S1: Pseudo ternary phase diagram of the SEME systems, Figure S2: Illustration of particle size distribution of blank and SEME, Figure S3: Shear sweep test, Figure S4: Peak hold experiment, Figure S5: Oscillatory (strain sweep) measurements, Figure S6: Skin penetration study by MPM-FLIM technique. Figure S7: Cell cycle histograms after RB treatment, Table S1: List of the SEME compositions, Table S2: Physicochemical properties of blank SEMEs.
