# Supplementary material for: Development of an Oil-in-Water Self-Emulsifying Microemulsion for Cutaneous Delivery of Rose Bengal: Investigation of Anti-Melanoma Properties
Source: Pharmaceutics. 2020 Oct 5;12(10):947. doi: 10.3390/pharmaceutics12100947 (PMC7600403; doi:10.3390/pharmaceutics12100947)
Supplement: Supplementary file 1 [file pharmaceutics-12-00947-s001.pdf]

pharmaceutics

# Supplementary Material: Development of an Oil-in-Water Self-Emulsifying Microemulsion for Cutaneous Delivery of Rose Bengal: Investigation of Anti-Melanoma Properties

**Farzaneh Forouz, Maryam Dabbaghi, Sarika Namjoshi, Yousuf Mohammed, Michael S. Roberts and Jeffrey E. Grice**

## 1. Composition of SEMEs

The compositions and percentages of SEMEs constituents are listed in Table S1.

**Table S1.** List of the SEME compositions.

| Rose Bengal Incorporated SEME Ingredients (*v/v* %) | | | | | | |
|---|---|---|---|---|---|---|
| | Oil Phase | S-Mix | | Aqua Phase | | Active |
| | | Surfactant | Co-surfactant | | | |
| Emulsion | Labrafac | Labrasol | Transcutol | PG | Water | RB |
| SEME+PG | 5 | 38.2 | 12.6 | 6.7 | 7.5 | 30 |
| SEME-PG | 5 | 39 | 13 | 0 | 13 | 30 |

| Blank SEME Ingredients (*v/v* %) | | | | | | | |
|---|---|---|---|---|---|---|---|
| | Oil Phase | S-Mix | | Aqua Phase | | Active | HLB of S-Mix |
| | | Surfactant | Co-surfactant | | | | |
| Emulsion | Labrafac | Labrasol | Transcutol | PG | Water | RB | |
| SEME + PG | 7.14 | 54.57 | 18 | 9.57 | 10.72 | 0 | 8.38 |
| SEME−PG | 7.14 | 55.71 | 18.57 | 0 | 18.58 | 0 | 7.42 |

## 2. Phase Diagrams of SEMEs

The process of self-emulsification was visually observed for the rate of emulsification and to determine the phase diagram region of the produced emulsions. The visual properties recorded against the addition of the applied surfactant mix in Ternary phase diagrams (Figure S1).

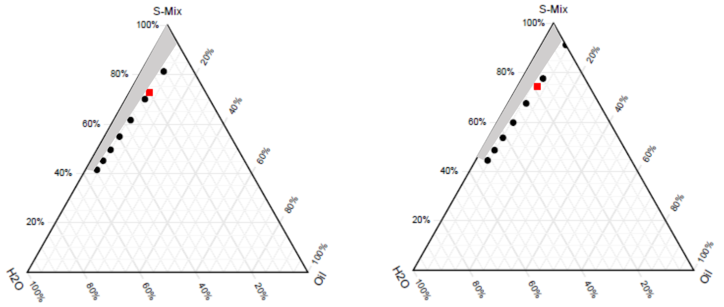


SEME + PG    SEME-PG

**Figure S1.** Pseudo ternary phase diagram of the SEME systems.

Phase diagrams were constructed by titration of oil/surfactant-mixtures (S-Mix) with water at room temperature. The Oil in Water microemulsion borders were reached when the phase separation occurred, and the solution turned turbid. The shaded areas within the dot points represent clear Oil in Water (O/W) microemulsions. The composition percentages of the microemulsion were selected within the isotropic region which are listed in the table and RB was incorporated into this formulation. The red dots show the SEME concentrations used in this study (Figure S1). Many SEMEs could be prepared from the O/W microemulsion region of the phase diagram; however, it is important to use the appropriate surfactant-mix concentration (surfactant plus co-surfactant) and use lowest possible concentration of surfactant, allowing for the non-toxic in vitro application in cell culture.

SEMEs contain Labrafac PG, water, Labrasol Transcutol. Labrasol: Transcutol (S-Mix). The volume ratio of S-Mix and Oil was fixed at 3 to 1. They grey shaded areas represent the isotropic regions for O/W micro-emulsion formation in this system created by water titration.

## 3. Droplet Size and Zeta Potential Determination

The size distribution of the RB incorporated SEME was measured and compared with the Blank SEME (Figure S2).

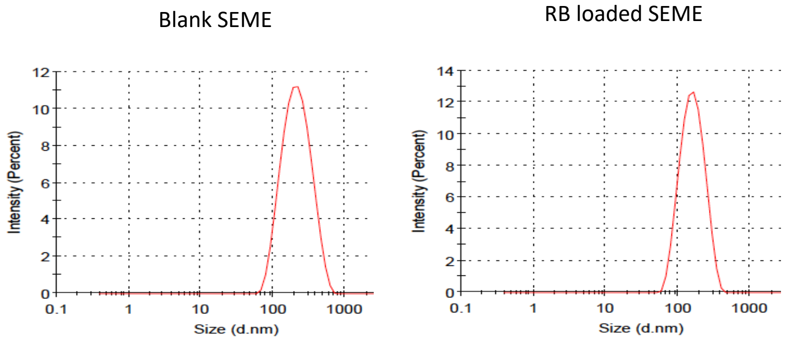


**Figure S2.** Illustration of particle size distribution of blank and SEME.

The graph shows (Figure S2) size distribution of the particles by intensity captured from the ZetaSizer. Incorporation of RB into the SEME-PG did not affect the size of the particles.

The results showed that the SEME systems formed micro size scale particles with Z-Averages of 115.3 ± 4.9 and 239.2 ± 4.5 (nm) for SEME−PG and SEME + PG respectively. The zeta potential of the blank microemulsions was measured. The charges were −0.0442 ± 3.3(mV) and −0.319 ± 9.8 for SEME + PG and SEME−PG respectively (Table S2).

**Table S2.** Physicochemical properties of blank SEMEs. Size and zeta potential were measured by Dynamic Light Scattering. pH was measured with pH meter. Results are shown as mean ± standard deviation. $n = 3$.

| Self-Emulsifying Micro Emulsion | Size (nm) | *PDI | Zeta potential (mv) | HLB of S-Mix | pH | Appearance |
|---|---|---|---|---|---|---|
| **SEME + PG** | 239.2 ± 4.5 | 0.38 ± 0.04 | −0.044 ± 3.3 | 8.38 | 5.37 | Transparent |
| **SEME−PG** | 115.3 ± 4.9 | 0.40 ± 0.02 | −0.319 ± 9.8 | 7.42 | 5.02 | Transparent |

*PDI: Polydispersity index.

In this study, PDI of less than 0.5 was defined as acceptable because the active drug (RB) is mainly loaded into the aqueous phase of the both SEME systems. The polydispersity indices of our microemulsions are in the range of 0.3 to 0.4, which could indicate a narrow deviation of average size for these systems.

## 4. Rheological Measurements

The shear sweep test is one of the fundamental tests for characterizing the rheological behaviour of the developed topical formulations. This test was performed to measure the apparent viscosity as a function of shear stress (Figure S3A. The shear stress was set to increase from 0.01 to 100 Pa with 10 points per decade. Also, the shear rate was plotted versus shear stress (Figure S3B).

In the peak hold experiment, the viscosity changes are evaluated at a constant shear stress during a defined period of time. Under the steady shear condition, the viscosity of Newtonian materials remains constant over the time period, the opposite of non-Newtonian materials. A series of peak hold experiments were performed with increasing applied shear stress (from 1 to 6 Pa) and time (5 and 10 min) (Figure S4). As it can be the viscosity of both formulations was constant after applying different shear stresses over different periods of time.

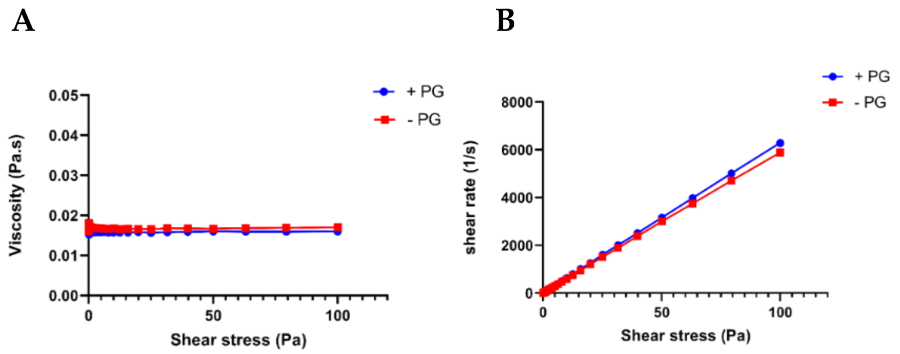


**Figure S3.** Shear sweep test. (**A**) apparent viscosity as a function of shear stress. (**B**) the shear rate versus shear stress

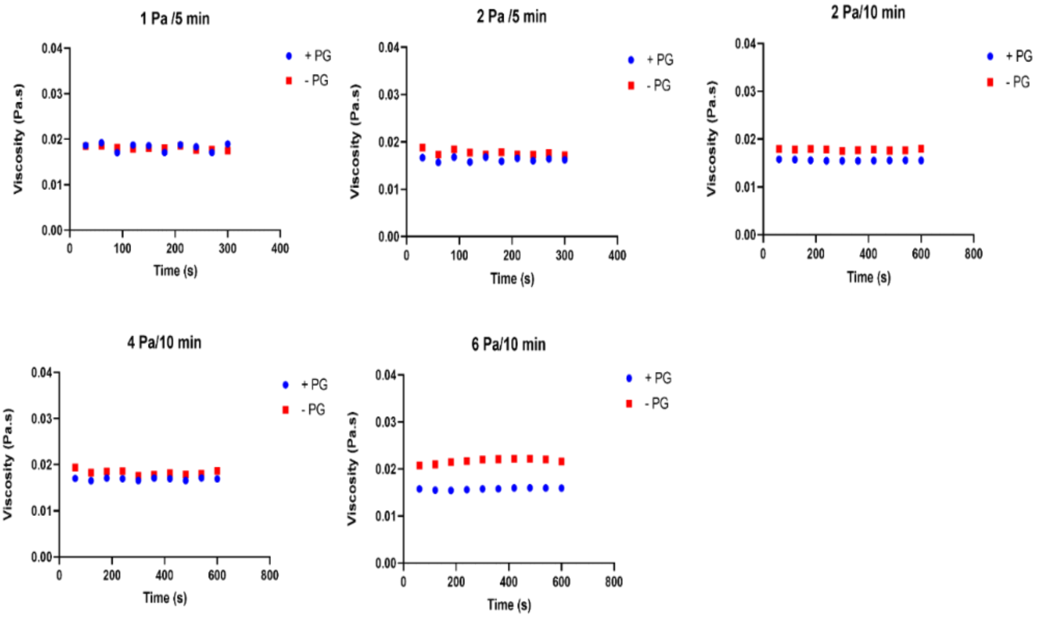


**Figure S4.** Peak hold experiment.

Oscillatory (strain sweep) measurements were performed over the range of 0.01 to 10,000% strain, with 5 points per decade (Figure S5). The measurements were performed by applying a constant 6.283 angular frequency (rad/s) in log mode. The storage modulus (G′), loss modulus (G″) and dynamic viscosity were calculated using a computer program supplied by TA Instruments.

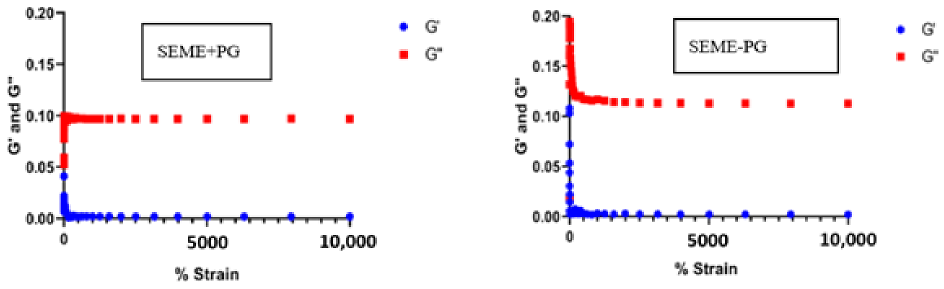


**Figure S5.** Oscillatory (strain sweep) measurements.

## 5. Effect of SEME on Skin Penetration of RB

RB skin delivery from microemulsion and aqueous solution was examined by MPM-FLIM and images from different layers of skin were captured (Figure S6A). Freshly excised human full thickness skin was incubated in 37 °C water bath with RB/W and RB/SEME for 24 h. To avoid treatment leakage from the skin sides, skins were secured in Franz Cells for the duration of treatment. Figure S6B shows normalized intensity of RB analysis result from each layer.

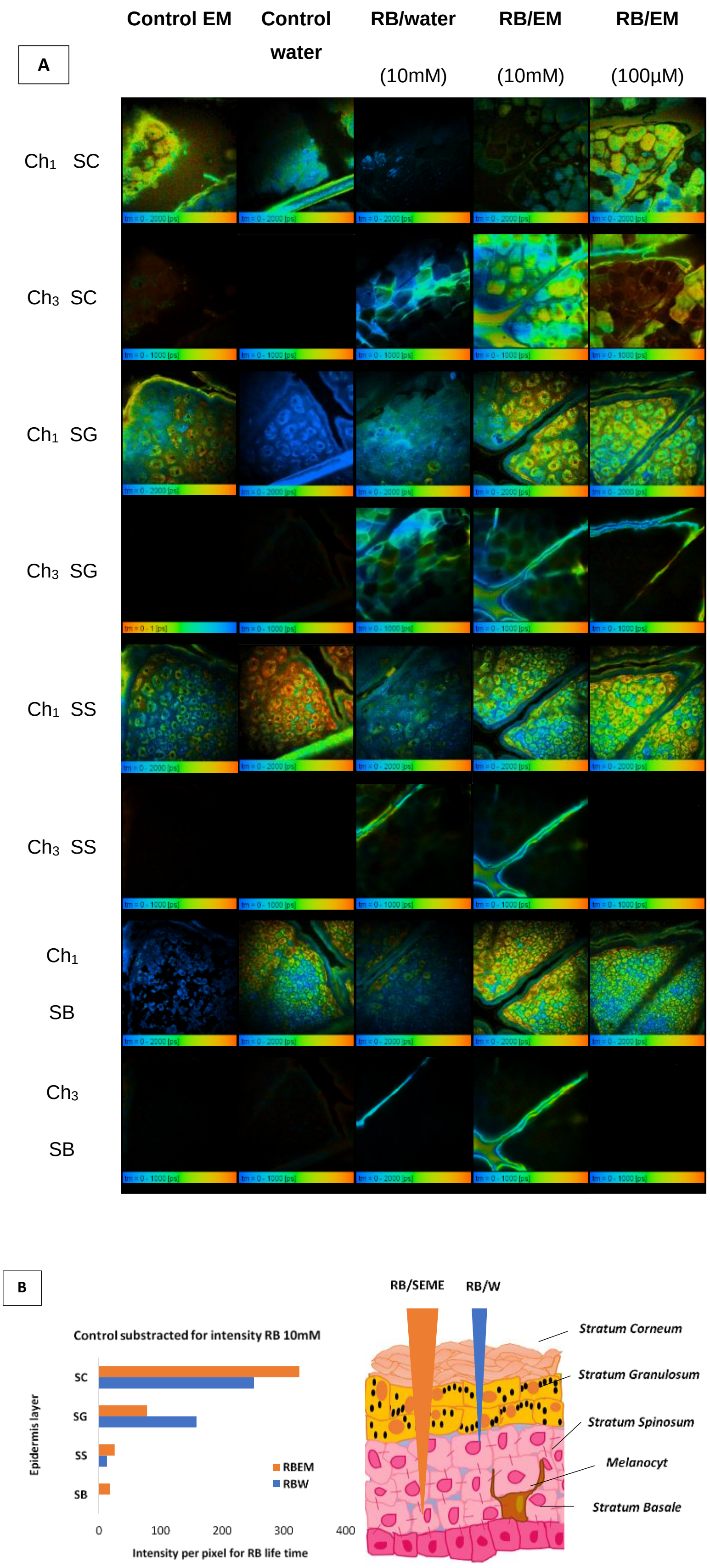


**Figure S6.** Skin penetration study by MPM-FLIM technique after exposure to the RB microemulsion and aqueous solution. (**A**) MPM-FLIM images from different layers of skin. (**B**) Normalized intensity of RB analysis result from each layer

Intensity of RB delivered in two delivery systems: Water and SEME-PG are captured using MPM-FLIM method. Freshly excised human full thickness skin was incubated in 37 °C water bath with RB/W and RB/SEME for 24 h. To avoid treatment leakage from the skin sides, skins were secured in Franz Cells for the duration of treatment. (A) Images from different layers from MPM-FLIM. (B) Normalized intensity of RB analysis result from each layer. SC: *Stratum Corneum*, SG: *Stratum Granulosum*, SS: *Stratum Spinosum*, and SB: *Stratum Basale*.

## 6. Cell Cycle Analysis by Flow Cytometry

After determining that RB decreased cell viability of human skin melanoma cells in a dose-dependent manner, the mechanism of cell death was investigated by flow cytometry. As RB-induced cell death was evident upon exposure to concentrations >50 µM, the cells were treated with 50-µM RB in water, SEME+PG and SEME-PG for 24 h for cell cycle analysis.

Histogram analysis of the sub $G_0G_1$ phase (Figure S7, region shown by asterisks) showed increases in apoptotic cell populations when cells were treated with RB-loaded SEMEs compared to the RB aqueous solution. Melanoma cells showed increases in apoptotic populations at various levels. However, equal dosage treatments did not impact normal keratinocytes and fibroblasts. This selective cell death mechanism could be a key for an effective antimelanoma drug.

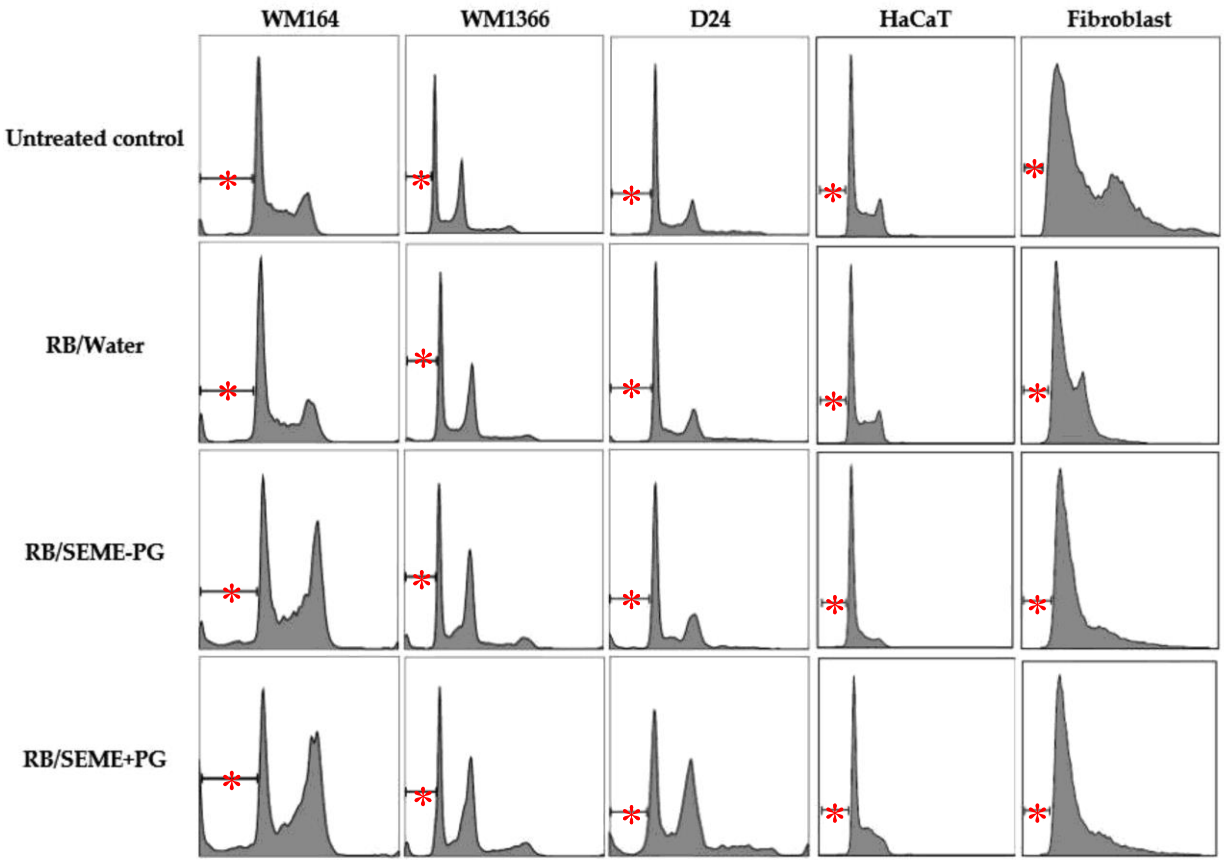


**Figure S7.** Cell cycle histograms after RB treatment. RB induces apoptosis in melanoma cells. Flow cytometry histograms of apoptosis assays by the PI method. Cells were treated with 50 µM of RB incorporated in different delivery systems for 24 h. The Sub$G_0G_1$ region (shown by asterisks) indicates the presence of apoptotic cells induced in melanoma cells treated with RB incorporated in SEMEs. The percentages of apoptotic cell populations in cancerous and non-cancerous cells are shown in Table 4 of the manuscript. The greatest apoptotic cell population was seen in the WM164 cells treated with RB loaded in a SEME containing PG.
